# Cardiac Tumors: A Brief Commentary

**DOI:** 10.3389/fpubh.2014.00264

**Published:** 2014-12-05

**Authors:** Leonardo Roever, Antonio Casella-Filho, Paulo Magno Martins Dourado, Elmiro Santos Resende, Antônio Carlos Palandri Chagas

**Affiliations:** ^1^Heart Institute (InCor), University of São Paulo Medical School, São Paulo, Brazil; ^2^Federal University of Uberlândia, Uberlândia, Brazil; ^3^Faculty of Medicine of ABC, Santo André, Brazil

**Keywords:** cardiac, tumors, myxoma, echocardiography, heart tumors

## Abstract

Patients with cardiac tumors may present with cardiovascular related or constitutional symptoms, but more often than not a cardiac mass is discovered incidentally during an imaging examination performed for an unrelated indication. Cardiac myxoma is generally considered to be a surgical emergency. Echocardiography, including the transesophageal approach, is the most important means of diagnosis; computed tomography and magnetic resonance imaging. The clinical presentation has changed, and the management of cardiac myxoma now needs to be reviewed.

## Introduction

It is called tumor any abnormal growth, whether it be cancerous or non-cancerous. Tumors originating from the heart are called primary tumors and can occur in any of its tissues. They can be cancerous or non-cancerous and are rare. Secondary tumors originate elsewhere in the body often the lung, breast, blood, or skin and then spread to the heart (1–3).

Secondary tumors are 30–40 times more common than primary, but still are considered unusual. Cardiac tumors may not cause symptoms or may produce a potentially lethal cardiac dysfunction, mimicking other diseases. Examples of such disorders include sudden heart failure, sudden onset of arrhythmias, and sudden drop in blood pressure due to bleeding in the pericardium (4–6).

Cardiac tumors are difficult to diagnose both being relatively uncommon, as its symptoms are similar to those of many other disorders. To arrive at a diagnosis, it is necessary that the physician has evidence of their presence. For example, if a person has cancer elsewhere in the body, but seeks medical attention because of symptoms related to cardiac dysfunction, the professional may suspect the presence of a cardiac tumor (5, 6). However, if a cardiac mass represents a tumor, its etiology can often be determined by considering four factors: (1) the histology-based likelihood; (2) the age of the patient at time of presentation; (3) the tumor location; and (4) non-invasive tissue characterization.

A PubMed, Europubmed search was performed until July 2014 using the terms “cardiac tumors” in combination with the terms “heart tumors,” “myxomas,” or “cardiac myxomas.”

## Myxomas

Myxoma is a non-cancerous tumor, and usually has an irregular shape and a gelatinous consistency. Half of all primary cardiac tumors are myxomas. Three quarters of myxomas located in the left atrium, the heart chamber that receives oxygen-rich blood from the lungs. Generally, the left atrial myxomas originate from a pedicle and can swing freely with the blood flow, like a ball attached to a wire. When oscillating, myxomas can move into and out of the mitral valve next the way between the left atrium and left ventricle. Cardiac myxomas are usually solitary and develop in the atria, 75% originating in the left atrium and 15–20% in the right atrium. They characteristically arise from or near the interatrial septum at the border of the fossa ovalis membrane. They occur in all age groups, most frequently between the third and sixth decades. Women are more commonly affected. Cardiac myxomas range in size from 1 to 15 cm in diameter (7–10).

This oscillation can block and unblock the valve continuously, so that the blood flow is stopped and restarted intermittently. In the standing position, the person may faint or episodes of pulmonary congestion and difficulty breathing, as the force of gravity causes the tumor to move downwards until the valve opening the position reduced the symptoms (11).

The tumor can damage the mitral valve and, consequently, a back flow of blood occurs through this opening, with the production of a murmur that the doctor listens with a stethoscope. Based on the characteristics of the murmur, the doctor considers whether the breath is the result of a flow caused by an injury arising out of the tumor, a rare cause or a more common cause of rheumatic heart disease. Fragments of a myxoma or blood clots that form on the surface of the myxoma may detach, move to other organs, and clog blood vessels in these locations. Symptoms depend on the blocked vessel: obstruction of a cerebral blood vessel can cause a stroke; obstruction of a pulmonary vessel can cause pain and bloody sputum (12). The clinical features of myxomas, like most cardiac tumors, are determined by their location, size, and mobility. Most patients present with one or more features of the triad of embolism, intracardiac obstruction, and constitutional symptoms. Dyspnea may occur secondary to atrioventricular valve obstruction. Other symptoms of myxomas include fever, weight loss, fingers and feet cold and sore when exposed to cold (Raynaud’s phenomenon), anemia, low platelet count (because platelets are involved in blood clotting), and symptoms suggestive of severe infection. Additionally, a myxoma can obstruct blood flow in the heart. Often they grow from a pedicle (resembling a mushroom) and are able to swing freely with the flow of the blood. This oscillating motion can obstruct blood flow through the mitral valve, the passageway between the left atrium and left ventricle. Non-cancerous less common cardiac tumors, such as fibroids and rhabdomyomas can grow directly from the cells of the fibrous tissue of the heart and myocardial cells. Rhabdomyomas, the second most common type of primary tumor, develop in childhood or early adolescence, often associated with a rare childhood disease called tuberous sclerosis. Other primary cardiac tumors, such as primary cancer, are extremely rare and, for them, there is no satisfactory treatment. Children with these tumors have a life expectancy of <1 year. Several tests are used in the diagnosis of cardiac tumors. Often, echocardiography (test that uses ultrasound waves to investigate the internal structures of the heart) is used for the delineation of tumors. The ultrasonic waves can pass through the chest wall or the inside of the esophagus transesophageal (10, 11).

A catheter inserted through a vein to the heart can be used to inject substances outlining a heart tumor on radiographs; but this procedure is less frequently needed. Computed tomography (CT) and magnetic resonance imaging (MRI) are also used. If a tumor is detected, a small sample can be removed through a special catheter; the sample will then be used to identify the type of tumor, which will help in selecting the appropriate treatment (11–13).

Echocardiography provides limited but useful information regarding tissue characteristics, including echogenicity of the mass and whether calcification is present. Vascularity can also be assessed using color flow Doppler and echocardiographic contrast. Strain imaging also has potential in identifying the non-contractile nature of masses such as fibromas. CT provides information regarding vascularity by contrast enhancement, presence of calcification, and presence of fat. Magnetic resonance imaging also provides information regarding vascularity, presence of fat, degree of tissue edema, and possibly iron content (14–23).

An isolated non-cancerous primary cardiac tumor can be removed by surgery, which generally cures the patient. The doctors do not treat primary tumors when there are many gifts, or treat tumors that are so great as to preclude removal. Although cardiac tumors are rare, they are being increasingly recognized antemortem, permitting earlier diagnosis and treatment. The most likely etiology of a cardiac mass is a thrombus or vegetation. In the current era, for benign cardiac tumors, an early diagnosis and appropriate treatment is not only possible but often curative.

## Conflict of Interest Statement

The authors declare that the research was conducted in the absence of any commercial or financial relationships that could be construed as a potential conflict of interest.
